# Ethical Aspects of Physician Decision-Making for Deprescribing Among Older Adults With Dementia

**DOI:** 10.1001/jamanetworkopen.2023.36728

**Published:** 2023-10-03

**Authors:** Jonathan D. Norton, Chan Zeng, Elizabeth A. Bayliss, Susan M. Shetterly, Nicole Williams, Emily Reeve, Matthew K. Wynia, Ariel R. Green, Melanie L. Drace, Kathy S. Gleason, Orla C. Sheehan, Cynthia M. Boyd

**Affiliations:** 1 Johns Hopkins University School of Medicine, Baltimore, Maryland; 2 Institute for Health Research, Kaiser Permanente Colorado, Aurora; 3 Department of Family Medicine, University of Colorado School of Medicine, Aurora; 4 Centre for Medicine Use and Safety, Faculty of Pharmacy and Pharmaceutical Sciences, Monash University, Melbourne, Victoria, Australia; 5 Quality Use of Medicines and Pharmacy Research Centre, Clinical and Health Sciences, University of South Australia, Adelaide, South Australia; 6 University of Colorado Center for Bioethics and Humanities, Anschutz Medical Campus, Aurora; 7 Department of Internal Medicine, University of Colorado School of Medicine, Aurora; 8 Department of Health Policy and Management, Colorado School of Public Health, Aurora; 9 RCSI Hospitals Group, Connolly Hospital, Dublin, Ireland

## Abstract

**Importance:**

Physicians endorse deprescribing of risky or unnecessary medications for older adults (aged ≥65 years) with dementia, but there is a lack of information on what influences decisions to deprescribe in this population.

**Objective:**

To understand how physicians make decisions to deprescribe for older adults with moderate dementia and ethical and pragmatic concerns influencing those decisions.

**Design, Setting, and Participants:**

A cross-sectional national mailed survey study of a random sample of 3000 primary care physicians from the American Medical Association Physician Masterfile who care for older adults was conducted from January 15 to December 31, 2021.

**Main Outcomes and Measures:**

The study randomized participants to consider 2 clinical scenarios in which a physician may decide to deprescribe a medication for older adults with moderate dementia: 1 in which the medication could cause an adverse drug event if continued and the other in which there is no evidence of benefit. Participants ranked 9 factors related to possible ethical and pragmatic concerns through best-worst scaling methods (from greatest barrier to smallest barrier to deprescribing). Conditional logit regression quantified the relative importance for each factor as a barrier to deprescribing.

**Results:**

A total of 890 physicians (35.0%) returned surveys; 511 (57.4%) were male, and the mean (SD) years since graduation was 26.0 (11.7). Most physicians had a primary specialty in family practice (50.4% [449 of 890]) and internal medicine (43.5% [387 of 890]). A total of 689 surveys were sufficiently complete to analyze. In both clinical scenarios, the 2 greatest barriers to deprescribing were (1) the patient or family reporting symptomatic benefit from the medication (beneficence and autonomy) and (2) the medication having been prescribed by another physician (autonomy and nonmaleficence). The least influential factor was ease of paying for the medication (justice).

**Conclusions and Relevance:**

Findings from this national survey study of primary care physicians suggests that understanding ethical aspects of physician decision-making can inform clinician education about medication management and deprescribing decisions for older adults with moderate dementia.

## Key Points

**Question:**

Do physicians prioritize different ethical considerations when considering deprescribing medications for older adults (aged ≥65 years) with dementia?

**Findings:**

In this national survey study of 890 primary care physicians, 9 factors influencing deprescribing decisions for older adults with dementia were prioritized. When a medication may increase risk of an adverse drug event or have limited benefit as a treatment, physicians are sensitive to concerns that deprescribing would worsen symptoms, are more reluctant to deprescribe a medication started by someone else, and less concerned about the cost of medication.

**Meaning:**

Results of this survey study suggest that managing symptoms and well-being and working with other clinicians are priorities for clinicians managing medications for older adults with dementia.

## Teaser

This survey study of primary care physicians assesses the prioritization of different ethical considerations when considering deprescribing medications for older adults with dementia.

## Introduction

Deprescribing has been defined in several studies^1,2,3^ as “the physician-supervised process of reducing or stopping a medication that is inappropriate or no longer necessary.” It is a component of clinical management that occurs at the juncture of ethical, pragmatic, and patient-centered care, as it directly reflects (often changing) goals of care and requires shared decision-making with patients and family members.^4^ Clinicians may experience ethical tensions around deprescribing decisions because deprescribing is perceived as an intentional action of discontinuing ongoing treatment.^4^ For example, recommending medication cessation to minimize harm in the face of limited treatment benefit (nonmaleficence) may be perceived as threatening patient autonomy when a patient continues to be interested in maximal disease management, or addressing the cost of unnecessary medications (to patients or the system) may raise questions of treatment equity among historically underrepresented and undertreated populations (justice).

Further, these ethical dilemmas related to optimal prescribing arise ubiquitously for clinicians treating people living with dementia who are particularly vulnerable to serious harm from polypharmacy (adverse drug events or drug interactions), treatment burden, and cognitive changes from adverse effects.^5,6,7,8,9^ The clinical management decisions for this population require ethically nuanced and pragmatic shared decision-making aligned with the patient’s goals of care, which can be further complicated by how to best respect patient autonomy in the context of impaired decision-making and how to consider benefits and risks to caregivers’ well-being stemming from managing symptoms for patients.^10^

Clinicians have little evidence to guide deprescribing decisions due to a clinical research culture focused on generating evidence to inform treatment intensification and methodologic limitations to conducting deprescribing investigations at scale. Without guidelines, clinicians may feel alone in their decision-making and have expressed reservations about stopping or reducing medicines. Articulated areas of uncertainty include changing medications started by other clinicians and having insufficient evidence on tapering and stopping medications.^11,12,13,14,15,16,17,18,19,20^ Similarly, deimplementation frameworks for cancer treatments acknowledge the difficulty in applying strategies to stop treatment when there is a lack of evidence to cease use and suggest the ethical and legal issues should be explored first in the absence of evidence and guidance.^21^

Understanding the influences on clinician decision-making in the context of potential ethical tensions and patient-centered pragmatic decision-making may help prepare clinicians for deprescribing discussions with their patients. In addition, understanding common deprescribing practices may inform the research agenda and support clinicians in undertaking thoughtful deprescribing until a formal evidence base becomes available.

This study aimed to understand influences on physician deprescribing decision-making. To help guide development of deprescribing guidance and clinician education, we surveyed a national sample of primary care physicians who care for older adults (aged ≥65 years) on perceived influences affecting their deprescribing decisions for persons with cognitive impairment.

## Methods

### Study Design and Sample

We mailed a self-administered 24-question survey entitled “Understanding Physician Deprescribing Decisions” (eMethods in Supplement 1) to a random sample of 3000 US physicians specializing in family medicine, general, internal, or geriatric medicine listed in the American Medical Association (AMA) Physician Masterfile (2020-2021). Physicians were randomized into equal groups to receive 1 of 2 survey versions presenting a clinical scenario in which the physician would like to deprescribe a medication for an older adult with moderate dementia either because (1) “There is evidence for increased risk of a serious acute adverse drug event (eg, fall or fracture) in this population if the drug is continued” (increased risk of adverse drug event [ADE] scenario), or (2) “There is no evidence of benefit from this medication in the older adult population with dementia” (limited benefit scenario). Information on demographic characteristics and practice were obtained from the AMA database including medical degree type (MD vs DO), primary specialty, census region, Federal Information Processing Standard (FIPS) code, sex, and year of graduation from medical school. We used the geographic FIPS code to match the corresponding 2013 Rural-Urban Continuum Codes to specify rural, urban, and suburban practice locations.^22,23^ We followed the American Association for Public Opinion Research (AAPOR) reporting guideline, and both institutional review boards from Kaiser Permanente Colorado and The Johns Hopkins University approved this study. Completion of the survey served as informed consent per a statement on the survey cover page.

### Survey Development

The survey was designed around 2 common scenarios in which clinicians may consider deprescribing: situations in which treatment presented an increased risk of an ADE and situations in which treatment would produce limited benefit (Table 1). We focused on the vulnerable population of older adults with moderate dementia taking 5 or more chronic medications who cannot live independently but can perform activities of daily living and who can safely be left alone for brief periods of time. Within these scenarios and this population, we presented respondents with a series of factors reflecting barriers to deprescribing that also illustrated biomedical ethical principles.^24,25^ We sought to evaluate the perceived barriers (biggest and smallest) influencing a physician’s decision to deprescribe a chronic medication within the given scenario. Factors were informed by previous research by several authors of the present study investigating physicians’ experiences with deprescribing among adults with cognitive impairment.^26^ The survey was pilot tested by 10 primary care physicians to confirm clarity of instructions and understanding of questions (these physicians were excluded from participating in the final survey). Based on pilot testing, we decoupled the 2 clinical scenarios into separate surveys.

**Table 1.** Clinical Scenarios and Bioethical and Pragmatic Factors for Best-Worst Scaling Questions^a^

Concept (illustrative bioethical domain)	Factor
Ease of paying for medication (justice)	The family reports that purchasing the medication does not pose a financial burden.
Fear patient’s/family’s perception as “giving up” on care (autonomy)	The family says that they see stopping medications as “giving up” on the patient.
More time required to discuss deprescribing (justice)	Discussing medication discontinuation will take more time than I have available at this visit.
Concern about cultural mistrust of care (justice)	The patient’s/family’s cultural background and societal history may include mistrust of health care.
Treatment of risk factor that may affect future health (beneficence)	This medicine treats a risk factor and so might improve their health in the future.
Patient/family reports symptomatic benefit from medication (beneficence/autonomy)	The patient/family reports that the medication helps a troublesome symptom (such as insomnia, reflux, or nausea).
Concern stopping medication risks clinical instability (nonmaleficence)	The patient is stable, and you are worried something bad could happen if you stop the medication.
Concern about achieving quality metric (beneficence/justice)	This medication is used as treatment to achieve a quality metric.
Medication prescribed by another physician (autonomy/nonmaleficence)	The medication was prescribed by a physician in a different specialty who still sees the patient.

^a^ Deprescribing scenarios: (1) increased risk of ADE: “There is evidence for increased risk of a serious acute adverse drug event (eg, fall or fracture) that could happen at any time if this drug is continued,” and (2) limited benefit: “There is no evidence of benefit for this medication in the older adult dementia population.”

We used the best-worst scaling (BWS) method to assess respondents’ rankings of perceived barriers to deprescribing a medication in this population.^27,28,29^ The BWS method presents respondents with multiple sets of factors (choice sets) in varied combinations to compare choices. Respondents then make choices within each set to select the best (or most important) and worst (or least important) factors for the given situation. In this survey, 9 factors/phrases were presented as potential barriers for physicians to select as the biggest or smallest barrier. With the BWS method, a limited number of items can be included. Use of the principles when designing the survey ensured that we had a broad variety of potential barriers covered (eg, ensuring that there were items related to justice). Pilot testing the survey allowed us to iteratively identify the best word choice for the 2 scenarios and 9 factors used in the BWS choice sets (Table 1).

Best-worst scaling was chosen because the task is known to be easily understood by respondents, and because it asks the respondent to make comparisons over multiple sets of questions instead of 1 single set.^27,28^ Additionally, the BWS method provides more discrimination in preferences than similar discrete choice experiment methods because it asks the respondent to select the worst factor in addition to the best factor, thus allowing for a more complete ranking of preferences than a partial ranking that comes from discrete choice experiments.^30^ For BWS choice sets, we used a balanced incomplete block design (calculated using Studio software, release 3.7, enterprise edition [SAS Institute Inc] to systematically repeat the 9 factors in different combinations of 3 over 12 blocks, in which each factor was compared with each of the other factors by appearing in 4 of the 12 blocks. The survey also assessed self-reported demographic characteristics, physician practice characteristics, and experiences with deprescribing medications.

### Survey Administration and Data Collection

Using the Dillman tailored design method for mailed surveys,^31^ a $5 bill was included in the first wave with a study introduction cover letter and a stamped return envelope. The first wave was sent in 2 batches (January 2021 and May 2021), and 2 follow-up waves were sent to nonresponders at 6-week intervals. Data collection occurred from January 15 until December 31, 2021.

### Statistical Analysis

Descriptive statistics were used to present self-reported demographic responses. We performed conditional logit regression to analyze the BWS questions because it is grounded in random utility theory and explains real-world choice behaviors.^28,30^ A phrase was assigned a value of −1 if it was chosen as the smallest barrier, 1 if chosen as the biggest barrier, and 0 if not chosen, while accounting for clustering by respondent and by choice set. The odds ratio (relative preference) for each phrase measures the respondents’ perceived importance of a barrier relative to a common reference, which was the smallest barrier to deprescribe the drug. We set the reference (smallest barrier) at 1, so a phrase/barrier with a relative preference of 2 means the phrase/barrier was perceived as twice as important compared with the reference phrase/barrier.

We also calculated individual best-minus-worst scores based on the difference between the number of times a factor was selected as the biggest barrier and the number of times it was selected as the smallest barrier. The range of scores was −4 to 4 as each phrase appeared equally in 4 of the 12 choice sets. We used best-minus-worst scores to examine variability in barrier ratings by estimating how often and how consistently each barrier was selected as the most or least important factor. For all analyses, we excluded respondents with missing or invalid choices. The answer to each block counted as 2 choices (biggest barrier and smallest barrier). Choices were designated as invalid if respondents chose more than 1 phrase as the biggest barrier or smallest barrier, if they chose the same phrase as the biggest barrier and smallest barrier, or if they did not choose a phrase as the biggest barrier or smallest barrier. All analyses were conducted using SAS version 9.4 (SAS Institute Inc).

## Results

### Respondent Characteristics

**Figure 1.**
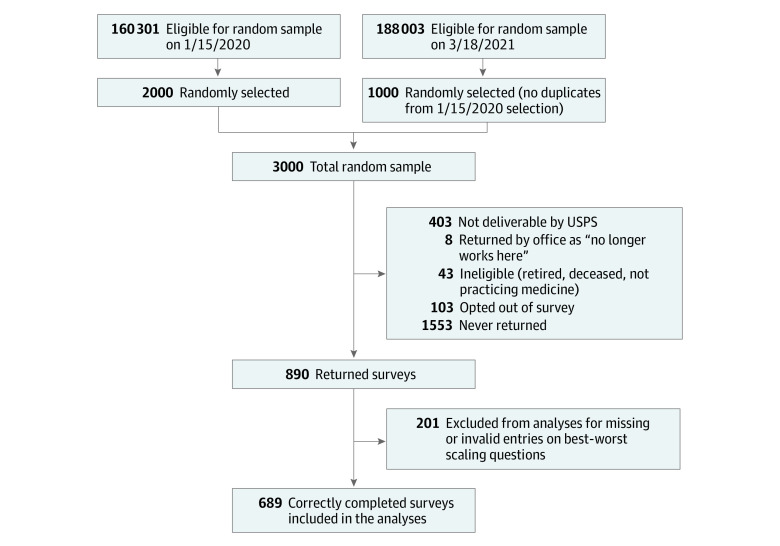
Survey Flowchart The eligibility specified for the 2 random samples were identical. The difference between the denominator for 2 random sample populations is due to the changes/updates to the American Medical Association Physician Masterfile at each date (ie, more physicians becoming eligible at a later date in 2021). Eligible physicians were primary care clinicians with a mailing address in any US state (territories not included) and could have a primary specialty in family practice, general practice, internal medicine, family practice–geriatrics, internal medicine–geriatrics, or geriatric psychiatry and/or a secondary specialty in the same primary specialty categories. USPS indicates United States Postal Service.

Of the 3000 invited physicians, 454 were excluded as undeliverable or ineligible (ie, retired, deceased, or no longer practicing medicine); of 2546 who were presumed to receive surveys, 890 physicians returned surveys (511 [57.4%] were male, and the mean [SD] years since graduation was 26.0 [11.7]), giving a response rate of 35.0% (890 of 2546) (Figure 1). Among respondents, 49.1% (437 of 890) responded to the increased risk of ADE scenario, and 50.9% (453 of 890) responded to the limited benefit scenario. We further excluded 201 surveys with missing and/or invalid entries on BWS questions, leaving 689 surveys with analyzable data. Survey responses were entered into REDCap.^32^ We conducted a data entry quality check on a random sample of 89 surveys (10%), and the data entry error rate was less than 0.0005 (1 of 2047).

The demographic characteristics of respondents vs nonrespondents from the AMA data set are shown in eTable 1 in Supplement 1, with slightly more respondents practicing in suburban and rural vs urban settings, having a little bit longer time in practice, and having family medicine as a specialty. Among respondents, 57.4% were male, 86.8% practiced in urban counties, and representation across census regions was relatively equal. Primary medical specialties were mostly family practice (50.4%) and internal medicine (43.5%). Self-reported characteristics of respondents with valid and complete BWS questions are shown in Table 2. We found no significant differences between the groups receiving either of the 2 clinical scenario surveys. Among respondents included in the analyses, 77.6% spent more than three-quarters of their time in direct patient care, 65.0% had more than 15 years of experience, and 82.5% reported less than one-quarter of their older patients had cognitive impairment. Thirty-four percent occasionally considered deprescribing chronic medications for their older adult patients with cognitive impairment during a typical visit, 41.6% reported being somewhat more likely to deprescribe compared with 5 years ago, and 22.5% reported having an experience in which they deprescribed a medication and the patient had a subsequent ADE that was related to the deprescribing.

**Table 2.** Self-Reported Characteristics of Respondents Included in Analyses

Characteristic	No. (%) (N = 689)^a^
Primary specialty	
Internal medicine	274 (39.8)
Geriatric medicine	30 (4.4)
Family medicine	363 (52.7)
Other/none selected	22 (3.2)
Years in practice since completing the last portion of medical training	
≤5 y	70 (10.2)
6-10 y	86 (12.5)
11-15 y	85 (12.4)
>15 y	447 (65.0)
Gender	
Male	389 (56.6)
Female	298 (43.4)
Proportion of your patients who are aged 65 y or older, %	
76-100	95 (13.9)
51-75	287 (42.0)
25-50	217 (31.8)
<25	84 (12.3)
Of your patients aged 65 y or older, proportion who have cognitive impairment, %	
>50	30 (4.4)
26-50	89 (13.1)
5-25	393 (57.7)
<5	169 (24.8)
Frequency of considering deprescribing a chronic medication during typical visits with patients with cognitive impairment	
Rarely	45 (6.6)
Occasionally	232 (34.0)
About half the time	139 (20.4)
Usually	169 (24.7)
Almost always	98 (14.3)
Compared with 5 y ago, likelihood to consider deprescribing a chronic medication for patients with cognitive impairment	
Much more	120 (17.6)
Somewhat more	284 (41.6)
About the same	240 (35.2)
Somewhat less	29 (4.3)
Much less	9 (1.3)
Proportion of time spent in direct patient care, %	
76-100	534 (77.6)
51-75	77 (11.2)
25-50	46 (6.7)
<25	20 (2.9)
No patient care	11 (1.6)
Proportion of practice time spent in outpatient care, %	
76-100	503 (73.1)
51-75	48 (7.0)
25-50	26 (3.8)
<25	29 (4.2)
No outpatient care	82 (11.9)
Outpatient practice setting	
Private practice	260 (37.7)
Academic practice	77 (11.2)
Multispecialty practice	112 (16.3)
Integrated delivery system	89 (12.9)
Other/none selected	151 (21.9)
Had any experiences in which you deprescribed a medication and the patient had a subsequent ADE	
Yes, related to the deprescribing	154 (22.5)
Yes, but I’m not sure it was related to the deprescribing	186 (27.2)
No	345 (50.4)

Abbreviation: ADE, adverse drug event.

^a^ No significant differences were observed between the 2 different survey groups (increased risk of ADE vs limited benefit), and the number of responses varied between 681 and 689 given that some respondents did not answer all questions.

### Ranking of Potential Ethical Barriers to Deprescribing

**Figure 2.**
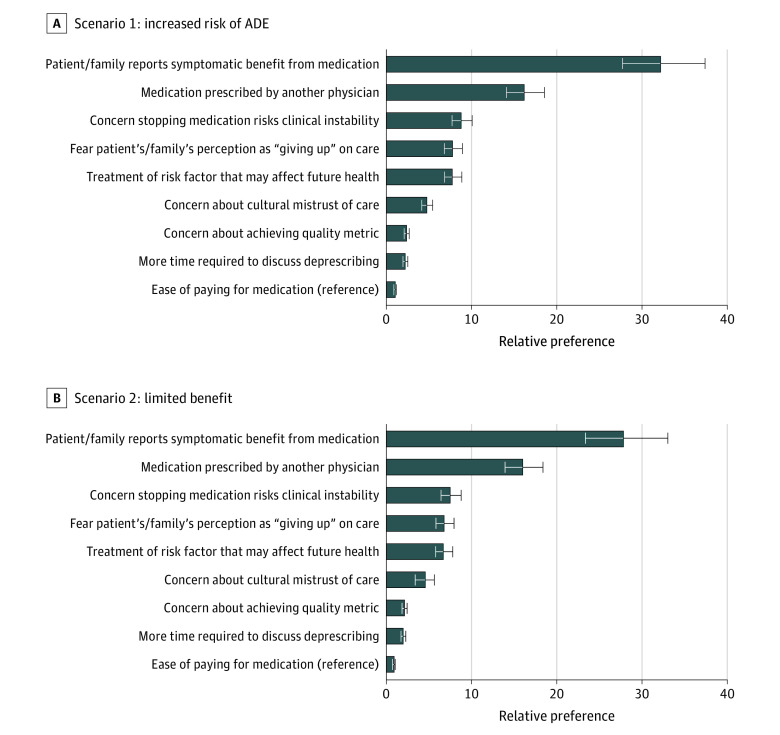
Relative Importance of 9 Barriers That May Influence a Physician’s Decision to Deprescribe a Medicine in an Older Adult With Moderate Dementia A phrase was assigned a value of −1 if chosen as the smallest barrier and 1 if chosen as the biggest barrier. We set the smallest barrier (perceived as the least important) as the reference at 1 so that a phrase with a relative preference of 2 means the phrase/barrier was perceived as twice as important compared with the reference phrase/barrier. Error bars indicate 95% CIs. ADE indicates adverse drug event.

Figure 2 illustrates the ranked odds of each barrier’s importance for each clinical scenario (increased risk of ADE and limited benefit to medication continuation). The 2 most important barriers and the least important barrier to deprescribing a medication were ranked similarly across both clinical scenarios, with “Patient/family reports symptomatic benefit from medication” being the most important barrier to deprescribing, followed by “Medication prescribed by another physician,” and the least important barrier was “Ease of paying for medication” (referent in both scenarios). The remaining barriers differed slightly in relative ranking of importance between the 2 scenarios (Figure 2; eTable 2 in Supplement 1).

### Variability of Barrier Choice Between Individuals

**Figure 3.**
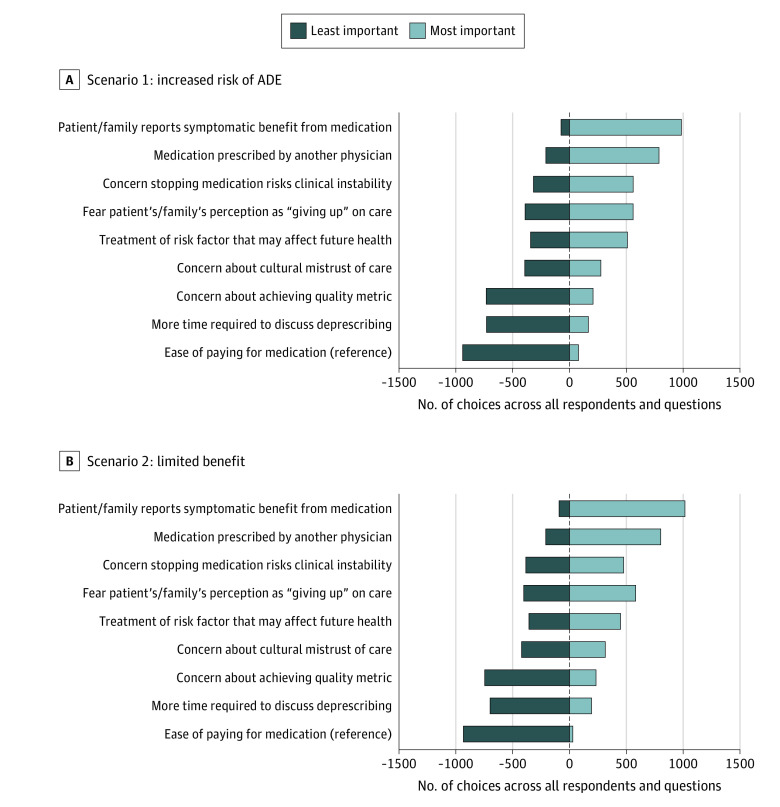
Variability of Perceived Barriers Between Individual Physicians This figure shows the number of times each phrase/barrier was chosen as the biggest and smallest barrier to deprescribing a medication (across the series of 12 sets of 3 phrases/barriers presented to each respondent) and ranked from biggest (right) to smallest barrier (left). Each phrase/barrier was presented 4 times to each participant, so the maximum number of times it could be chosen as the biggest or smallest barrier was 1368 (342×4) for scenario 1 and 1388 (347×4) for scenario 2. ADE indicates adverse drug event.

Figure 3 displays the number of times each barrier was chosen as the most and as the least important barrier to deprescribing a medication for both clinical scenarios, ranked from most important to least important. No single barrier was always chosen as the most or least important in either scenario. In the “increased risk of ADE” and “limited benefit” scenarios, 118 (34.5%) and 136 (39.2%) respondents, respectively, always chose “Patient/family reports symptomatic benefit from medication” as the most important barrier, and 104 (30.4%) and 95 (27.4%) respondents, respectively, always chose “Ease of paying for medication” as the least important barrier.

## Discussion

We conducted a national survey study exploring how primary care physicians prioritize barriers linked to several ethical and pragmatic considerations when making deprescribing decisions in caring for older individuals living with moderate dementia. To our knowledge, this is the first exploration of how ethical considerations affect physician deprescribing. We found that when a medication is known to have an increased risk of an ADE or has limited to no benefit as a treatment, physicians are particularly sensitive to patient or family member concerns about deprescribing (that is, autonomy). Physicians also appear to be reluctant to interfere with their colleagues’ medical management and deprescribe a medication started by someone else (autonomy and nonmaleficence) and are much less concerned about the financial impact of continuing or discontinuing a medication (justice). Overall, barriers related to the ethical principle of justice appeared to be less important barriers than those related to beneficence, nonmaleficence, and autonomy.

In both clinical scenarios, our findings that physicians view symptom management as the most important barrier to deprescribing aligns with a previous national physician survey of cardiologists, general internists, and geriatricians trying to deprescribe cardiovascular medications in older adults.^33^ Goyal et al^33^ found that regardless of specialty, a “patient’s reluctance toward deprescribing” was the second most common barrier. Our study highlights that addressing patient preferences (ie, considering autonomy) will be a key factor in the development of deprescribing resources and guidelines. How this can be conducted when a patient cannot communicate those preferences in later and more advanced stages of Alzheimer disease and related dementia requires further research. Green et al^1,25^ surveyed older adults living without Alzheimer disease and related dementia on their preferences of language and communication from clinicians on deprescribing statins or sedative hypnotic medications and found that language should convey the nature of the shared decision with the patient and/or caregiver, and the rationale of deprescribing should center on the risk of adverse effects. While our study focuses on older adults living with moderate dementia and on deprescribing medications in general, taken together, these findings further support the need for deprescribing guidance for physicians underpinned by ethical principles to support complex decision-making.

Our finding that physicians view medications being prescribed by other specialists/colleagues as a major barrier to deprescribing bolsters similar findings in other recent physician and primary care clinician surveys on deprescribing cardiovascular medications^33^ and opioids or benzodiazepines in older adults,^34^ as well as from previous qualitative research in community practice settings in the US^11,16,18^ and outside the US.^12,13,14,15,17,19,20^ We considered this barrier to be related to the principles of autonomy (of the specialists) and nonmaleficence, as it was felt that altering the prescribing of another clinician could lead to negative consequences. Prescribers could be choosing the path of least resistance because of system-level barriers such as health care system fragmentation,^15^ deference to colleagues’ clinical decision-making, time required to coordinate care with other clinicians, and adequate time for shared decision-making with patients.^12,16,33,34^ Personal physician-level barriers such as lack of confidence or education to deprescribe medications could also be a factor.^34^ Further investigation is needed to understand how to best educate and empower physicians on how to engage in deprescribing conversations with specialists.

Our finding that physicians consider the financial impact of continuing or discontinuing a medicine as the least important barrier to deprescribing may provide an alternative entry point to starting conversations about deprescribing with their patients and may require clinician education. As highlighted by Hung et al,^7^ deprescribing conversations might start with discussing medications that risk an ADE or medications with no evidence of benefit, but patients may also wish to address prescription costs and medication burden. Up to 68% of patients report that they spend a lot of money on their medications or that having to pay less for medicines would play a role in their willingness to deprescribe.^35^ Physicians may want to assess their patients’ out-of-pocket costs for their medications before starting a deprescribing conversation so that they can achieve better goal-aligned and person-centered care.

Overall, our findings have implications for physicians and future research. By linking barriers to ethical principles, physicians will be able to reflect on what might be behind the barriers they experience and explore whether they are allowing a single principle, such as autonomy, to dominate their decision-making. This work may inform research aimed at developing guidance on how ethical principles can be used as a framework to support decision-making around deprescribing, especially when there is a lack of evidence to inform the likely benefits and harms of deprescribing certain medications in complex situations. Given the finding that factors related to nonmaleficence do appear to play a large role in deprescribing decision-making, future research could explore how this could be used as a trigger for deprescribing. In qualitative studies,^36^ a common barrier to deprescribing is that physicians don’t want to “rock the boat.” Reeve et al^4^ proposed that physicians view deprescribing as an act, with continuation viewed as not acting (omission).^4^ Therefore, the negative outcomes from deprescribing are given a greater moral weight than the negative outcomes from continuation. This indicates that if physicians see the decision to continue as also active, the importance of nonmaleficence that we found in this study could also help promote deprescribing.

Our work may also inform future designs of deimplementation interventions to deprescribe inappropriate medications. For example, a common physician-level characteristic that affects deimplementation is past negative experiences.^37^ In our survey, 22.5% of respondents reported they were sure their patients experienced an adverse event after deprescribing. To improve physician-level factors in deprescribing interventions, future research should explore the relationship between the severity and frequency of negative past events of deprescribing and the physician’s willingness to deprescribe.^37^

### Strengths and Limitations

Our national survey study of physicians had some key strengths. First, the survey used BWS methods to elicit respondent preferences in a complete ranking as compared with Likert scale methods. Second, the survey was conducted and framed around a particularly relevant and vulnerable patient population that is at great risk of adverse effects of polypharmacy. Third, our study focused on primary care for older adults with Alzheimer disease and related dementia, which has been identified as a research priority and clinical need.^38^

Given the importance of symptom management as a barrier to deprescribing, there is a need for greater access to nonpharmacological approaches^39,40,41,42^ to manage the symptoms that lead to the use of medications with possible ADEs in older adults with dementia. Strategies that address communication between clinicians around the use of medications^43^ may be beneficial to address the barrier of medications being prescribed by another physician. Emerging tools to help clinicians address deprescribing in clinical practice now include tapering guidelines for benzodiazepines and deprescribing algorithms, brochures, and whiteboard videos for multiple drug classes.^44,45,46,47,48,49^ Patient priority care has delineated a framework in which primary care clinicians can communicate with a specialist in a patient goal–directed way that provides a platform for deprescribing when consistent with the patient’s goals.^43^

Our study also had several limitations. First, the study was conducted during the COVID-19 pandemic, and this may have influenced physicians’ responses as they relate to the care of individuals living with Alzheimer disease and related dementia. Second, our response rate was in the middle range for contemporary physician surveys, which raises the potential for response bias. However, given similar response rates to other survey studies conducted recently,^50^ this more likely reflects a COVID-19 pandemic effect on response rates in general rather than response bias to this particular study, and we did not see meaningful differences in demographic characteristics between respondents and nonrespondents. Also, survey delivery could only be confirmed on return of a completed survey; the delivery of US mail has seen many pandemic-related delays, making it difficult to assess the number of surveys successfully delivered. As such, our response rate may be an underestimation. Third, we had a some missing responses in the BWS and excluded respondents from the analysis (eTable 3 in Supplement 1).

## Conclusions

Understanding ethical aspects of physician decision-making can inform clinician education about medication management and deprescribing decisions for older adults with moderate dementia. This research highlights that clinician education on deprescribing should include shared decision-making to prioritize goals of care and patient symptom management and that clinician-to-clinician communication is essential. Further research on the most effective tools and strategies is needed. Addressing these barriers through deprescribing strategies and educational initiatives will be important to reduce medication-related harm in older adults living with dementia.

## Notes

Supplement 1. **eMethods.** Survey Instrument Content **eTable 1.** Characteristics of Respondents and Non-Respondents **eTable 2.** Data for Figure 2 **eTable 3.** Comparison of Respondent Characteristics of Completed Surveys vs Surveys With Missing Entries From the Best-Worst Scaling Questions

Supplement 2.
Data Sharing Statement
