# Supplementary material for: Ethical Aspects of Physician Decision-Making for Deprescribing Among Older Adults With Dementia
Source: JAMA Netw Open. 2023 Oct 3;6(10):e2336728. doi: 10.1001/jamanetworkopen.2023.36728 (PMC10548310; doi:10.1001/jamanetworkopen.2023.36728)
Supplement: Supplement 1. — eMethods. Survey Instrument Content eTable 1. Characteristics of Respondents and Non-Respondents eTable 2. Data for Figure 2 eTable 3. Comparison of Respondent Characteristics of Completed Surveys vs Surveys With Missing Entries From the Best-Worst Scaling Questions [file jamanetwopen-e2336728-s001.pdf]

Supplemental Online Content

Norton JD, Zeng C, Bayliss EA, et al. Ethical aspects of physician decision-making for deprescribing among older adults with dementia. *JAMA Netw Open*. 2023;6(10):e2336728. doi:10.1001/jamanetworkopen.2023.36728

eMethods. Survey Instrument Content

eTable 1. Characteristics of Respondents and Non-Respondents

eTable 2. Data for Figure 2

eTable 3. Comparison of Respondent Characteristics of Completed Surveys vs Surveys With Missing Entries From the Best-Worst Scaling Questions

This supplemental material has been provided by the authors to give readers additional information about their work.

© 2023 Norton JD et al. *JAMA Network Open*.

eMethods. Survey Instrument Content

Physician Survey to Understand Deprescribing Decisions

This survey explores how physicians make decisions about "deprescribing," or stopping or decreasing the dose of chronic medicines. The survey uses a clinical scenario about deprescribing for an older adult with moderate dementia.

- The survey takes about 10 minutes. There are no right or wrong answers. We want your honest opinions and experiences.
- **Your response is very important:** Deprescribing is a critical issue for patients, clinicians and policy makers. You have been selected to represent many other physicians.
- Your completion of this survey will serve as your consent to be in this research study.
- Your answers are anonymous and cannot be traced back to you. The number on the survey serves only to track responses to avoid repeat mailings.

We greatly appreciate your time and effort.

<u>Section A</u>

1. What proportion of your patients are aged 65 or older?

   ☐ 76-100%

   ☐ 51-75%

   ☐ 25 – 50%

   ☐ Less than 25%

2. Of your patients age 65 or older, how many have cognitive impairment?

   ☐ More than 50%

   ☐ 26 – 50%

   ☐ 5 - 25%

   ☐ Less than 5%

3. How often do you <u>consider</u> deprescribing a chronic medication during typical visits with patients with cognitive impairment?

   ☐ Rarely

   ☐ Occasionally

   ☐ About half the time

   ☐ Usually

   ☐ Almost always

© 2023 Norton JD et al. *JAMA Network Open*.

4. Compared to 5 years ago, are you <u>now more or less likely to consider</u> deprescribing a chronic medication for patients with cognitive impairment?

   - ☐ Much more
   - ☐ Somewhat more
   - ☐ About the same
   - ☐ Somewhat less
   - ☐ Much less

© 2023 Norton JD et al. *JAMA Network Open*.

Section B

Instructions

Imagine you are having an office visit with an older patient (65+):

- with moderate dementia
- who cannot live independently but can perform activities of daily living
- who takes more than 5 chronic medications
- who can safely be left alone for brief periods of time during the day

You would like to deprescribe a medication that this patient has been taking **because there is evidence for increased risk of a serious acute adverse drug event (e.g. fall or fracture) in this population** if this drug is continued.

For each question, we will show you 3 factors that could affect your decision to deprescribe the medication **at this visit**. Within each block of 3 items, please choose the one factor that would be the **biggest barrier to deprescribing** the medication and which would be the **smallest barrier**. For each block compare only the 3 options presented.

In the survey items for this section, we will repeat – in different combinations – the same 9 factors that may influence deprescribing decisions.

EXAMPLE Question – not a survey item:

**BIGGEST barrier to deprescribing (choose one)**	**Barriers to deprescribing a medication that you would like to deprescribe**	**SMALLEST barrier to deprescribing (choose one)**
☑	A new caregiver has accompanied the patient today and is not familiar with the patient's medications	☐
☐	The patient has moved into a skilled care facility	☑
☐	The patient just refilled a 90-day supply of medication	☐

In this example, the respondent felt that – of the choices offered—having a new caregiver was the biggest barrier to deprescribing the medication, and the patient moving into a skilled care facility was the smallest barrier to deprescribing the medication at this visit.

© 2023 Norton JD et al. *JAMA Network Open*.

The survey questions start here:

Question 5:

**<u>BIGGEST</u> barrier to deprescribing (choose one)**	**Barriers to deprescribing a medication that you <u>would like to deprescribe</u> due to:**<br><br>**<u>evidence for an increased risk of a serious acute adverse drug event (e.g. fall or fracture)</u> in older adults with dementia**	**<u>SMALLEST</u> barrier to deprescribing (choose one)**
☐	The patient/ family reports that the medication helps a troublesome symptom (such as insomnia, reflux or nausea).	☐
☐	Discussing medication discontinuation will take more time than I have available at this visit.	☐
☐	The patient is stable, and you are worried something bad could happen if you stop the medication.	☐

Question 6:

**<u>BIGGEST</u> barrier to deprescribing (choose one)**	**Barriers to deprescribing a medication that you <u>would like to deprescribe</u> due to:**<br><br>**<u>evidence for an increased risk of a serious acute adverse drug event (e.g. fall or fracture)</u> in older adults with dementia**	**<u>SMALLEST</u> barrier to deprescribing (choose one)**
☐	The medication is used to achieve a quality metric.	☐
☐	The medicine treats a risk factor and so might improve their health in the future.	☐
☐	Discussing medication discontinuation will take more time than I have available at this visit.	☐

Question 7:

**<u>BIGGEST</u> barrier to deprescribing (choose one)**	**Barriers to deprescribing a medication that you <u>would like to deprescribe</u> due to:**<br><br>**<u>evidence for an increased risk of a serious acute adverse drug event (e.g. fall or fracture)</u> in older adults with dementia**	**<u>SMALLEST</u> barrier to deprescribing (choose one)**
☐	The family says that they see stopping medications as 'giving up on' the patient.	☐
☐	The medication is used to achieve a quality metric.	☐
☐	The patient/ family's cultural background and societal history may include mistrust of health care.	☐

© 2023 Norton JD et al. *JAMA Network Open*.

Question 8:

**BIGGEST** barrier to deprescribing (choose one)	**Barriers to deprescribing a medication that you would like to deprescribe due to:**<br><br>**evidence for an increased risk of a serious acute adverse drug event (e.g. fall or fracture) in older adults with dementia**	**SMALLEST** barrier to deprescribing (choose one)
☐	The medicine treats a risk factor and so might improve their health in the future.	☐
☐	The patient/ family reports that the medication helps a troublesome symptom (such as insomnia, reflux or nausea).	☐
☐	The family says that they see stopping medications as 'giving up on' the patient.	☐

Question 9:

**BIGGEST** barrier to deprescribing (choose one)	**Barriers to deprescribing a medication that you would like to deprescribe due to:**<br><br>**evidence for an increased risk of a serious acute adverse drug event (e.g. fall or fracture) in older adults with dementia**	**SMALLEST** barrier to deprescribing (choose one)
☐	The medication was prescribed by a physician in a different specialty who still sees the patient.	☐
☐	The medicine treats a risk factor and so might improve their health in the future.	☐
☐	The family reports that purchasing the medication does not pose a financial burden.	☐

Question 10:

**BIGGEST** barrier to deprescribing (choose one)	**Barriers to deprescribing a medication that you would like to deprescribe due to:**<br><br>**evidence for an increased risk of a serious acute adverse drug event (e.g. fall or fracture) in older adults with dementia**	**SMALLEST** barrier to deprescribing (choose one)
☐	The patient/ family's cultural background and societal history may include mistrust of health care.	☐
☐	The patient is stable, and you are worried something bad could happen if you stop the medication.	☐
☐	The medicine treats a risk factor and so might improve their health in the future.	☐

© 2023 Norton JD et al. *JAMA Network Open*.

Question 11:

**BIGGEST** barrier to deprescribing (choose one)	**Barriers to deprescribing a medication that you would like to deprescribe due to:**<br><br>**evidence for an increased risk of a serious acute adverse drug event (e.g. fall or fracture) in older adults with dementia**	**SMALLEST** barrier to deprescribing (choose one)
☐	The medication was prescribed by a physician in a different specialty who still sees the patient.	☐
☐	The medication is used to achieve a quality metric.	☐
☐	The patient/ family reports that the medication helps a troublesome symptom (such as insomnia, reflux or nausea).	☐

Question 12:

**BIGGEST** barrier to deprescribing (choose one)	**Barriers to deprescribing a medication that you would like to deprescribe due to:**<br><br>**evidence for an increased risk of a serious acute adverse drug event (e.g. fall or fracture) in older adults with dementia**	**SMALLEST** barrier to deprescribing (choose one)
☐	The patient is stable, and you are worried something bad could happen if you stop the medication.	☐
☐	The family reports that purchasing the medication does not pose a financial burden.	☐
☐	The medication is used to achieve a quality metric.	☐

Question 13:

**BIGGEST** barrier to deprescribing (choose one)	**Barriers to deprescribing a medication that you would like to deprescribe due to:**<br><br>**evidence for an increased risk of a serious acute adverse drug event (e.g. fall or fracture) in older adults with dementia**	**SMALLEST** barrier to deprescribing (choose one)
☐	The family says that they see stopping medications as 'giving up on' the patient.	☐
☐	The family reports that purchasing the medication does not pose a financial burden.	☐
☐	Discussing medication discontinuation will take more time than I have available at this visit.	☐

© 2023 Norton JD et al. *JAMA Network Open*.

Question 14:

**BIGGEST** barrier to deprescribing (choose one)	**Barriers to deprescribing a medication that you would like to deprescribe due to:** <br>**evidence for an increased risk of a serious acute adverse drug event (e.g. fall or fracture) in older adults with dementia**	**SMALLEST** barrier to deprescribing (choose one)
☐	The patient is stable, and you are worried something bad could happen if you stop the medication.	☐
☐	The family says that they see stopping medications as 'giving up on' the patient.	☐
☐	The medication was prescribed by a physician in a different specialty who still sees the patient.	☐

Question 15:

**BIGGEST** barrier to deprescribing (choose one)	**Barriers to deprescribing a medication that you would like to deprescribe due to:** <br>**evidence for an increased risk of a serious acute adverse drug event (e.g. fall or fracture) in older adults with dementia**	**SMALLEST** barrier to deprescribing (choose one)
☐	Discussing medication discontinuation will take more time than I have available at this visit.	☐
☐	The medication was prescribed by a physician in a different specialty who still sees the patient.	☐
☐	The patient/ family's cultural background and societal history may include mistrust of health care.	☐

Question 16:

**BIGGEST** barrier to deprescribing (choose one)	**Barriers to deprescribing a medication that you would like to deprescribe due to:** <br>**evidence for an increased risk of a serious acute adverse drug event (e.g. fall or fracture) in older adults with dementia**	**SMALLEST** barrier to deprescribing (choose one)
☐	The family reports that purchasing the medication does not pose a financial burden.	☐
☐	The patient/ family's cultural background and societal history may include mistrust of health care.	☐
☐	The patient/ family reports that the medication helps a troublesome symptom (such as insomnia, reflux or nausea).	☐

© 2023 Norton JD et al. *JAMA Network Open*.

<u>Section C</u>

Please answer a few final questions. You are almost finished!

17. What is your primary specialty?

☐ Internal Medicine

☐ Geriatric Medicine

☐ Family Medicine

☐ Other____________

18. How many years have you been in practice since completing the last portion of your medical training (e.g. residency, fellowship)?

☐ 5 years or less

☐ Between 6 and 10 years

☐ Between 11 and 15 years

☐ More than 15 years

19. What is your gender?

☐ Female

☐ Male

☐ Other preferred designation __________________

20. What proportion of your time do you spend in direct patient care?

☐ 76-100%

☐ 51-75%

☐ 25 – 50%

☐ Less than 25%

☐ No patient care

21. What proportion of your practice time do you spend in outpatient care?

☐ 76-100%

☐ 51-75%

☐ 25 – 50%

☐ Less than 25%

☐ No outpatient care

© 2023 Norton JD et al. *JAMA Network Open*.

22. How would you describe your outpatient practice setting?

☐ Private practice

☐ Academic practice

☐ Multispecialty practice

☐ Integrated delivery system

☐ Other ____________

23. Have you had any experiences in which you deprescribed a medication and the patient had a subsequent adverse outcome?

☐ Yes, related to the deprescribing

☐ Yes, but I'm not sure it was related to the deprescribing

☐ No

24. Please share any comments about your experience.

____
____
____

This is the end of the survey. Thank you very much for your time and effort to complete it.

© 2023 Norton JD et al. *JAMA Network Open*.

eTable 1. Characteristics of respondents and non-respondents

Characteristic (From AMA Dataset)	Responder No. (%)	Non-Responder No. (%)	p-value[a]
Total	N=890 (34.9)	N=1,659 (65.1)	
Total with AMA data[b]	N=887 (34.8)	N=1,659 (65.2)	
**Male**	511 (57.6)	939 (56.6)	0.62
**Medical Degree**			
MD	762 (85.9)	1448 (87.3)	0.33
DO	125 (14.1)	211 (12.7)	
**Years Since Graduation – Mean (SD)**	26.0 (11.7)	24.8 (11.5)	0.008
**Primary Specialty**			0.005
Family Practice (FP)	449 (50.6)	744 (44.8)	
FP/Geriatric Med	3 (0.3)	6 (0.4)	
General Practice	21 (2.4)	41 (2.5)	
Internal Medicine (IM)	387 (43.6)	837 (50.5)	
IM/Geriatrics	25 (2.8)	23 (1.4)	
Psychiatry – Geriatrics	2 (0.2)	8 (0.5)	
**Census Region**			0.0005
Northeast	175 (19.7)	310 (18.7)	
South	218 (24.6)	328 (19.8)	
Midwest	268 (30.2)	632 (38.1)	
West	226 (25.5)	389 (23.4)	
**RUCC 2013**			
Urban (1-3)	770 (86.8)	1522 (91.7)	0.0002
Suburban (4-6)	80 (9.0)	103 (6.2)	
Rural (7-9)	37 (4.2)	34 (2)	

Abbreviations: AMA, American Medical Association; RUCC, Rural Urban County Codes.

[a] p value from Chi-square for all variables except ‘Years since graduation' which was compared by T-test.

[b] Three surveys were returned without the tracking number and could not be included in the descriptive analysis.

© 2023 Norton JD et al. *JAMA Network Open*.

eTable 2. Data for Figure 2

eTable 2a: Increased risk for ADE

Phrase Tested	Parameter Estimate (STE)	OR (95% CI)
Patient/Family reports symptomatic benefit from medication	3.47 (0.08)	32.29 (27.80, 37.49)
Medication prescribed by another physician	2.78 (0.07)	16.11 (14.02, 18.52)
Concern stopping medication risks clinical instability	2.17 (0.07)	8.72 (7.62, 9.97
Fear patient’s/family’s perception as ‘giving up’ on care	2.04 (0.07)	7.70 (6.74, 8.80)
Treatment of risk factor that may affect future health	2.03 (0.07)	7.65 (6.69, 8.74)
Concern about cultural mistrust of care	1.55 (0.07)	4.70 (4.12, 5.36)
Concern about achieving quality metric	0.81 (0.07)	2.26 (1.98, 2.57)
More time required to discuss deprescribing	0.75 (0.07)	2.12 (1.86, 2.42)
Ease of paying for medication (Reference)	reference	1 (reference)

eTable 2b: Limited Benefit

Phrase Tested	Parameter Estimate (STE)	OR (95% CI)
Patient/Family reports symptomatic benefit from medication	3.34 (0.07)	28.09 (24.29, 32.48)
Medication prescribed by another physician	2.66 (0.07)	14.30 (12.50, 16.36)
Fear patient’s/family’s perception as ‘giving up’ on care	1.92 (0.07)	6.82 (6.00, 7.75)
Treatment of risk factor that may affect future health	1.783 (0.07)	5.95 (5.24, 6.76)
Concern stopping medication risks clinical instability	1.781 (0.07)	5.94 (5.23, 6.75)
Concern about cultural mistrust of care	1.45 (0.06)	4.25 (3.74, 4.82)
More time required to discuss deprescribing	0.78 (0.06)	2.19 (1.93, 2.48)
Concern about achieving quality metric	0.76 (0.06)	2.13 (1.88, 2.42)
Ease of paying for medication (Reference)	reference	1 (reference)

Abbreviations: ADE, Adverse Drug Event; STE,Standard Error; OR,Odds Ratio; CI,Confidence Interval.

© 2023 Norton JD et al. *JAMA Network Open*.

eTable 3. Comparison of respondent characteristics of completed surveys vs surveys with missing entries from the best-worst scaling questions

Characteristic	Survey Complete n(%), total=689[a]	Survey Missing n(%), total=201[b]	p-value
**Survey type**			
Increased risk of ADE	342 (49.6%)	95 (47.3%)	0.554
Limited Benefit	347 (50.4%)	106 (52.7%)	
**Primary Specialty**			
Internal Medicine	274 (39.8%)	82 (40.8%)	<0.001
Geriatric Medicine	30 (4.4%)	4 (2.0%)	
Family Medicine	363 (52.7%)	78 (38.8%)	
Other/None selected	22 (3.2%)	37 (18.4%)	
**Years in practice since completing the last portion of medical training**			
5 years or less	70 (10.2%)	14 (8.0%)	0.442
Between 6 and 10 years	86 (12.5%)	16 (9.1%)	
Between 11 and 15 years	85 (12.4%)	25 (14.3%)	
More than 15 years	447 (65.0%)	120 (68.6%)	
**Gender**			
Female	298 (43.4%)	62 (35.8%)	0.072
Male	389 (56.6%)	111 (64.2%)	
**Proportion of patients that are aged 65 or older**			
76-100%	95 (13.9%)	24 (12.1%)	0.756
51-75%	287 (42.0%)	84 (42.4%)	
25 - 50%	217 (31.8%)	69 (34.8%)	
Less than 25%	84 (12.3%)	21 (10.6%)	
**Of patients aged 65 or older, proportion that have cognitive impairment**			
More than 50%	30 (4.4%)	11 (5.5%)	0.623
26 - 50%	89 (13.1%)	24 (12.1%)	
5 - 25%	393 (57.7%)	122 (61.3%)	
Less than 5%	169 (24.8%)	42 (21.1%)	
**Frequency of considering deprescribing a chronic medication during typical visits with patients**			
Rarely	45 (6.6%)	19 (9.5%)	0.551
Occasionally	232 (34.0%)	64 (32.0%)	
About half the time	139 (20.4%)	43 (21.5%)	
Usually	169 (24.7%)	51 (25.5%)	
Almost always	98 (14.3%)	23 (11.5%)	
**Likelihood to consider deprescribing a chronic medicine compared to 5 years ago**			
Much more	120 (17.6%)	46 (23.1%)	0.258[c]
Somewhat more	284 (41.6%)	73 (36.7%)	

© 2023 Norton JD et al. *JAMA Network Open*.

eTable 3. Comparison of respondent characteristics of completed surveys vs surveys with missing entries from the best-worst scaling questions

Characteristic	Survey Complete n(%), total=689[a]	Survey Missing n(%), total=201[b]	p-value
About the same	240 (35.2%)	65 (32.7%)	
Somewhat less	29 (4.3%)	10 (5.0%)	
Much less	9 (1.3%)	5 (2.5%)	
**Proportion of time spent in direct patient care**			
76-100%	534 (77.6%)	143 (82.2%)	
51-75%	77 (11.2%)	21 (12.1%)	
25 - 50%	46 (6.7%)	6 (3.4%)	0.211[c]
Less than 25%	20 (2.9%)	4 (2.3%)	
No patient care	11 (1.6%)	0 (0.0%)	
**Proportion of practice time spent in outpatient care**			
76-100%	503 (73.1%)	140 (80.0%)	
51-75%	48 (7.0%)	4 (2.3%)	
25 - 50%	26 (3.8%)	6 (3.4%)	0.152
Less than 25%	29 (4.2%)	5 (2.9%)	
No outpatient care	82 (11.9%)	20 (11.4%)	
**Outpatient practice setting**			
Private practice	260 (37.7%)	69 (34.3%)	
Academic practice	77 (11.2%)	13 (6.5%)	
Multispecialty practice	112 (16.3%)	37 (18.4%)	0.004
Integrated delivery system	89 (12.9%)	16 (8.0%)	
Other/No selected	151 (21.9%)	66 (32.8%)	
**Had any experiences deprescribing a medication and the patient had a subsequent adverse outcome**			
Yes - related to the deprescribing	154 (22.5%)	39 (22.7%)	
Yes - but I'm not sure it was related to the deprescribing	186 (27.2%)	43 (25.0%)	0.842
No	345 (50.4%)	90 (52.3%)	

Abbreviations: ADE, Adverse Drug Event

[a] The number of responses varied between 681 and 689 given that some respondents did not answer all questions.

[b] The number of responses varied between 172 and 201 given that some respondents did not answer all questions.

[c] p-value from Exact Fischer's Test

© 2023 Norton JD et al. *JAMA Network Open*.
