# Supplementary material for: Ethical Aspects of Physician Decision-Making for Deprescribing Among Older Adults With Dementia
Source: JAMA Netw Open. 2023 Oct 3;6(10):e2336728. doi: 10.1001/jamanetworkopen.2023.36728 (PMC10548310; doi:10.1001/jamanetworkopen.2023.36728)
Supplement: Supplement 2. — Data Sharing Statement [file jamanetwopen-e2336728-s002.pdf]

Data Sharing Statement

Norton. Ethical Aspects of Physician Decision-Making for Deprescribing Among Older Adults With Dementia. *JAMA Netw Open*. Published October 03, 2023.
doi:10.1001/jamanetworkopen.2023.36728

Data
Data available: No
